# Network Pharmacology-Based Strategy for Predicting Active Ingredients and Potential Targets of Gegen Qinlian Decoction for Rotavirus Enteritis

**DOI:** 10.1155/2020/2957567

**Published:** 2020-07-29

**Authors:** Peicheng Zhong, Lijun Song, Mengyue Gao, Xiaotong Wang, Wenpan Tan, Huanqian Lu, Qian Lan, Zuyi Zhao, Wenchang Zhao

**Affiliations:** ^1^School of Pharmacy, Guangdong Medical University, Dongguan 523808, Guangdong, China; ^2^Guangdong Key Laboratory for Research and Development of Natural Drugs, Guangdong Medical University, Zhanjiang 524023, Guangdong, China; ^3^School of Mathematics, Sun Yat-Sen University, Guangzhou 510275, Guangdong, China

## Abstract

**Materials and Methods:**

In this study, a network pharmacology-based strategy was used to elucidate the mechanism of GGQLD for the treatment of RVE. Oral bioavailability and drug-likeness were taken as the judgment criteria to search the active ingredients of GGQLD in traditional Chinese medicine systems pharmacology database and analysis platform (TCMSP). The affinity between protein and ingredients was further determined using the similarity ensemble approach to find the corresponding targets. According to the genes related to enteritis in GeneCards database, the key targets were screened by intersections between drug and disease targets. And the therapeutic mechanism was predicted using the protein-protein interactions (PPIs), the Gene Ontology (GO), and the Kyoto Encyclopedia of Genes and Genomes (KEGG) database, which was verified by detecting calcium ion concentration with the fluorescent probe.

**Result:**

130 active ingredients were screened from GGQLD, including (R)-canadine, moupinamide, formononetin, and other flavonoids. They act on a total of 366 targets, which is mainly distributed in the biological process of hormone binding or signaling pathways of neuroactive ligand receptor interaction, serotonergic synapse, and calcium signaling pathway. Furthermore, serotonin receptors, adrenergic receptors, cholinergic receptors, and dopamine receptors in the enteric nervous system may be the key targets of RVE treatment by GGQLD.

**Conclusion:**

This study demonstrated that the potential mechanism that GGQLD can effectively improve the symptoms of RVE may depend on the regulation of calcium ions, serotonin, and gastrointestinal hormone ion that could mutually affect the intestinal nervous system.

## 1. Introduction

Traditional Chinese medicine (TCM), which has been widely used in clinical practice in China for thousands of years, takes multimedicinal materials compatibility for treating complex diseases [1]. And TCM formulas could be considered a complex system that consists of hundreds of chemical compounds, which exert synergistic, mutual assistance therapeutic action with fewer side effects on the complex giant system of the human body [2]. Thus, it presents a serious challenge to explain the mechanism of action of TCM formulas.

Network pharmacology is a systematic analytical method based on the interaction network of diseases, genes, protein targets, and drugs to elucidate the mechanism of drug action [3]. With the help of computational network pharmacology, it could explain TCM formulas' acting characteristics of integrity, synergy, and dynamic in a holistic view and affords new sights into the multicomponent and multitargeted therapeutics of TCM. Therefore, network pharmacology holds a powerful and promising tool for analyzing TCM formulas.

GGQLD, a four-herb Chinese medicine formula first described 1900 years ago, is composed of Pueraria lobate, Scutellaria baicalensis, Coptis chinensis, and Glycyrrhiza uralensis. It has definite curative effect on RVE [4, 5], which is characterized by diarrhea, vomiting, fever, and so on. More than 200,000 infant deaths by RV are noted annually without specific drugs in clinic [6]. But the complex composition of GGQLD makes it difficult to conduct molecular mechanistic research on it in depth.

**Figure 1:**
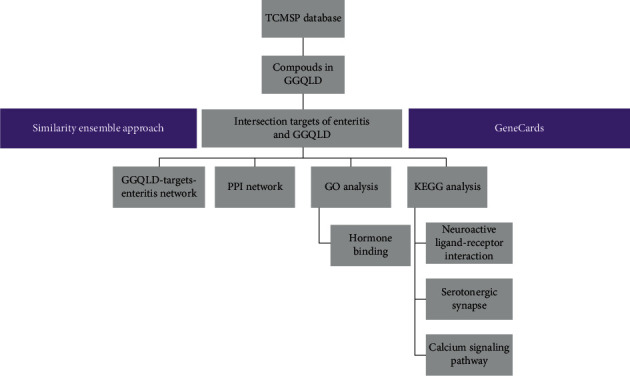
Flowchart of the systems pharmacology approach for hypothetical mechanism of GGQLD in treating RVE by ingredients collection, targets identification, and network analysis.

In this study, network pharmacology strategy was adopted to study the mechanism of GGQLD against RVE. The active ingredients of GGQLD and their targets intersected with disease genes were identified to investigate network relationships between drugs, targets, and diseases through network pharmacology (Figure 1).

## 2. Materials and Methods

### 2.1. Reagent and Materials

Caco-2 cells were from Wuhan university cell bank (Wuhan, China). RV-WA was from the immunology institute of the Third Military Medical University (Chongqing, China). Hoechst dye was purchased from Solarbio Company (Beijing, China). Fura-3 fluorescence was purchased from Beyotime Company (Shanghai, China). The Pueraria lobate, Scutellaria baicalensis, Coptis chinensis, and Glycyrrhiza uralensis were purchased from Dongguan Sinopharm Group (product batch number: 20190501, Dongguan, China). High glucose medium and fetal bovine serum were purchased from Gibco Company (USA).

### 2.2. Database Construction

#### 2.2.1. Compound Database Construction

We obtained information of GGQLD active ingredients from the traditional Chinese medicine systems pharmacology database and analysis platform (TCMSP) (http://sm.nwsuaf.edu.cn/lsp/tcmsp.php), which is a comprehensive TCM database for network pharmacology and also provides ideal information of the ADME (absorption, distribution, metabolism, and excretion) properties of natural compounds [7]. With TCMSP, the active ingredients of GGQLD were mainly filtered through the criteria of oral bioavailability (OB) and drug-likeness (DL), which are important indicators to evaluate ADME properties through bioinformatics. The increase in OB can achieve maximum efficacy and minimal side effects [8]. DL has been widely used to screen compounds with undesirable properties [9]. The QSAR models based on multiple linear regression (MLR), partial least squares regression (PLS), and nonlinear support-vector machine regression (SVR) have good potential to predict OB [10]. For DL, it is used in the drug design after evaluating whether it is similar to the physicochemical property and structural characteristics of existing drugs, which refers to molecular weight, one-dimensional descriptors, two-dimensional profiles, three-dimensional variables, and total positive and negative charges. In general, we calculate the DL by the Tanimoto coefficient, a formula based on database [11]: *f(x,y)* *=* *xy/|x|*^*2*^ *+* *|y|*^*2*^ *−* *xy* (*X* represents the new numerator, and *y* represents the overall parameters of all ingredients in Drug-Bank database). In this study, active ingredients with OB index ≥30% and DL index ≥0.18 were selected as follow-up studies.

#### 2.2.2. Active Ingredients Targeted Protein and Genes Database Construction

To obtain more detailed multicomponent drug targets, the next essential step was to find the specific targets where the drug ingredients play a pharmacodynamic role [12]. With the development of systems biology and computational methods, the ways to predict molecular targets are more abundant and reliable [13]. Here, we used a technique called similarity ensemble approach (http://sea.bkslab.org/) to obtain protein targeted by active ingredients from GGQLD, which is based on the chemical similarity among their ligands to match protein. Although the ligands are similar in the structure, both of them do not have an identical ligand [14]. Before searching the target protein limited to Homo sapiens in similarity ensemble approach, we had to get the canonical SMILES of the active ingredients as search term through PubChem platform (https://pubchem.ncbi.nlm.nih.gov/). Finally, we further used UniProtKB database (http://www.uniprot.org/) to verify the standard protein name and obtain the corresponding gene name as targets, which is the authority for the functional information of proteins [15].

#### 2.2.3. RVE Disease Target Genes Database Construction

To maximize the total effective disease targets, we search them with the keyword “enteritis” in the database called GeneCards (https://www.genecards.org). From this database, we can obtain the relevant action enteritis-related genes of Homo sapiens, which included the all targets associated with RVE in particular.

### 2.3. TCM-Intersecting Targets-Disease Network Construction

The intersecting targets, displayed by Venn diagrams, were obtained between the targets of GGQLD ingredients and the RVE disease genes, which were used for the construction of PPI network and TCM-intersecting targets-disease network. First, based on the intersecting targets, PPI network was constructed to predict the interacting proteins through the STRING database (https://string-db.org/) with the multiple proteins function and the threshold of 0.95 interaction score. And the statistical interaction connectivity of proteins was used to determine the top 8 proteins. Next, in order to visualize the TCM-intersecting targets-disease network, we used Cytoscape software 3.7.1 to express the relationship between them in the form of a network diagram, where the nodes represented diseases, ingredients, or targets, and the lines represented nodes' associations.

### 2.4. Biological Function Analysis

Through R language software (3.4.4) and Biocon-ductor's biocLite.R, the above intersecting targets were performed to GO enrichment analysis (from the biological processes) and KEGG enrichment analysis. GO (http://geneontology.org/) is used to annotate the genes of different species from three independent ontologies including biological process, molecular function, and cellular component [16]. Furthermore, KEGG (http://www.genome.jp/kegg/) is a database that has access to specifically analyze the distribution of intersecting targets in the pathway [17]. Finally, *P* < 0.05 was selected as the standard of significant enrichment to obtain the biological processes and signaling pathways in which GGQLD participated in the anti-RVE pharmacological role. The Cytoscape software 3.7.1 was used to visualize and analyze their related targets and active ingredients interaction network.

### 2.5. Functional Validation by Detection of Calcium Ions

#### 2.5.1. Preparation of GGQLD and Cytotoxicity Test

15 g of Pueraria lobate, 9 g of Scutellaria baicalensis, 9 g of Coptis chinensis, and 6 g of Glycyrrhiza uralensis with 8 times weight of water were taken. Pueraria lobate was decocted for 20 min firstly, and the remaining herbs were decocted for a total of 30 min. Besides, the half-inhibitory concentration (IC50) of GGQLD to Caco-2 cells was measured by MTT colorimetry.

#### 2.5.2. Cell Culture and GGQLD Treatment

Caco-2 cells were routinely cultured in high-sugar DMEM medium containing 10% fetal bovine serum (containing 1% antipenicillin and streptomycin). It was placed in an incubator at 37°C and 5% carbon dioxide. After it grew to a monolayer, it was routinely digested. Then, it was inoculated on the laser confocal special dish according to the density of 1 × 10^5^ cells, which were divided into the control group, RV group, and GGQLD group. After 24 h of culture, the culture medium of the RV group and GGQLD group was removed. Then, 0.1 ml RV-WA with a virus titer of 10^−4^/0.1 ml (firstly incubated with 10 *μ*g/ml trypsin for 30 min at 37°C) and 0.9 ml serum-free DMEM culture medium were added, while the control group was added 1 ml serum-free DMEM culture medium. After 2 h of infection, the culture medium of the control group and RV group was removed and 2 ml serum-free DMEM culture medium was added for 24 h. But the GGQLD group was added 2 ml serum-free DMEM culture medium with 4.8 mg/ml of GGQLD.

#### 2.5.3. Determination of Intracellular Calcium Ion Intensity

Upon completion, changes in intracellular calcium ion intensity were measured using a laser confocal microscope (Leica SP8) and fura-3 fluorescence loading technology. The cells were incubated in serum-free DMEM culture solution containing 5 *μ*M fluo-3/AM fluorescent probe at 37°C for 30 min. The incubation solution was removed and then added 1 ml 5 *μ*g/ml Hoechst dye to be incubated for 5 min. Finally, the cells were washed twice with PBS buffer solution. Finally, the fluorescence intensity of calcium ions was measured with the laser confocal microscope at the excitation wavelength of 488 nm and the absorption wavelength of 515–565 nm.

## 3. Results

### 3.1. Composition Analysis

After searching, 142 active ingredients that passed the OB and DL filters were identified from the TCMSP database. However, only 130 of these ingredients showed the shared targets across disease genes (shown in Table 1), and the active ingredients with the number of intersecting targets >5 are listed in Table 2.

### 3.2. TCM-Intersecting Targets-Disease Network Analysis

**Figure 2:**
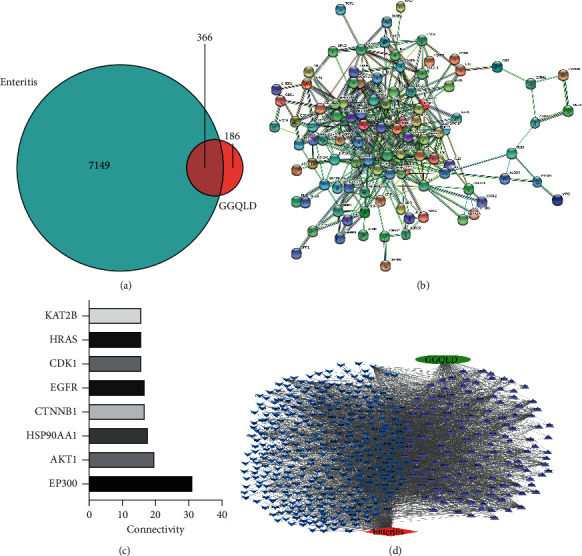
TCM-intersecting targets-disease network diagram: (a) intersecting targets of enteritis and GGQLD. There are 7149 enteritis-related targets (blue section) and 552 active ingredients targets (red section), with 366 intersections; (b) PPI network of intersecting targets; (c) genes with top 8 PPI network connectivities. The abscissa represents the number of connections with other genes, and the ordinate represents the name of the gene; (d) network of GGQLD (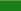), enteritis (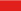), intersecting targets (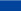), and active ingredients (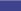) in molecular level. In the diagram, intersecting targets were represented by gene IDs and active ingredients were represented by the PubChem IDs.

Finally, we found 7149 enteritis-related targets and 552 GGQLD active ingredient targets, with 366 intersections (Figure 2(a)). The 366 intersecting targets were imported into the String database to obtain the PPI network targeting anti-RVE (Figure 2(b)). In this network, as shown in Figure 2(c), the top 8 genes with the highest node connectivity were EP300, AKT1, HSP90AA1, CTNNB1, EGFR, CDK1, HRAS, and KAT2B. Among them, EP300 has 31 connections with other genes, and 7 other genes have more than 15 connectivities. Finally, with the intersection genes as a hub, we constructed a network diagram of GGQLD-intersecting targets-active ingredients (represented by PubChem ID) RVE, which could clearly determine the relationship between them (Figure 2(d)).

### 3.3. Biological Functional Analysis

**Figure 3:**
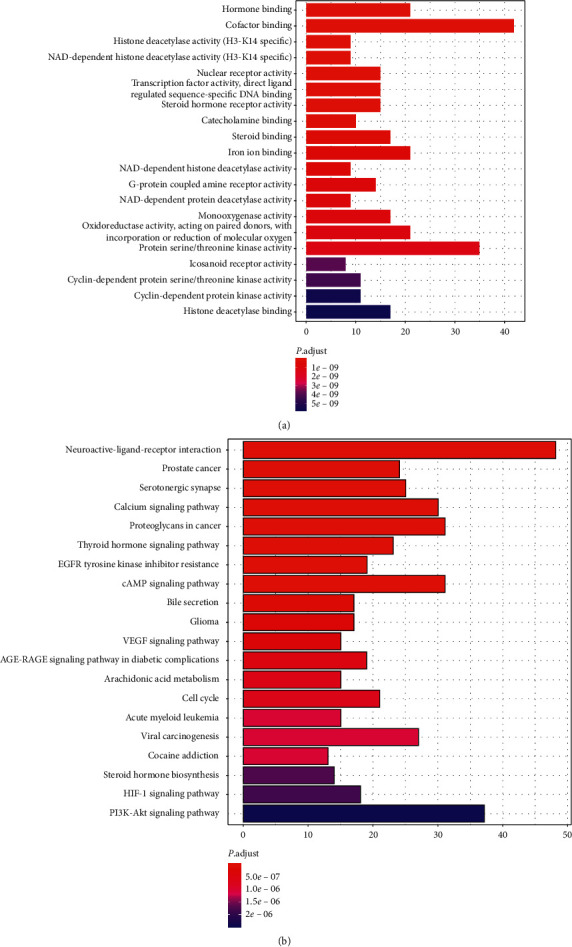
Biological function analysis of GGQLD anti-RVE targets: (a) GO enrichment analysis of the intersecting targets' biological process; (b) KEGG enrichment analysis of intersecting targets' signaling pathway.

In terms of biological processes, the intersecting targets demonstrate multiple functions (Figure 3(a)). The redder the color in the figure, the smaller the *P* value, and the stronger the representativeness. The most interesting one is hormone binding with ID 0042562. The function is interpreted as selectively and noncovalently interacting with any of the hormones to influence the metabolism or behavior of other cells with hormone-functional receptors.

In the signal pathway, its importance evaluation criteria were similar to the GO diagram (Figure 3(b)). The results showed the following pathways with reference value: neuroactive ligand-receptor interaction with ID hsa04080, serotonergic synapse with ID hsa04726, calcium signaling pathway with ID hsa04020, and so on. These pathways were concentrated in the related expression of neural active substances, especially in the serotonergic system.

### 3.4. Active Ingredients-Interaction Targets-Pathways Network Analysis

**Figure 4:**
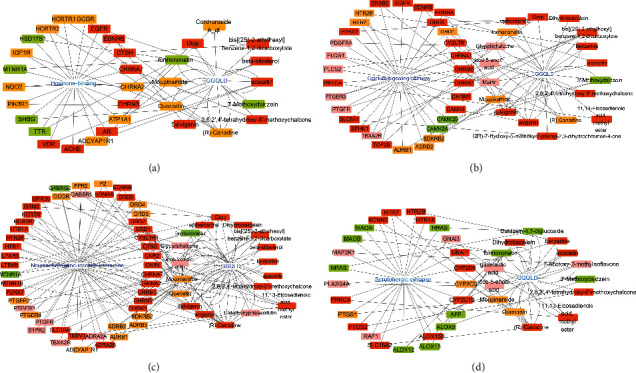
Network of interaction targets-active ingredients-pathways from the perspective of key function and pathways: (a) hormone binding; (b) calcium signaling pathway; (c) neuroactive ligand-receptor interaction; (d) serotonergic synapse (Pueraria lobate, Scutellaria baicalensis, Coptis chinensis, and Glycyrrhiza uralensis are showed by 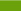, 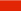, 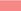, and 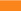).

Through Cytoscape software 3.7.1, the active ingredients and interaction targets related to the biological process horizon binding and the neuroactive ligand receptor interaction, serotonergic synapse, and calcium signaling pathway were visualized as the network (Figure 4). Among them, the active ingredients (R)-canadine, moupinamide, and formononetin, respectively, targeted 17, 17, and 11 targets. It is speculated that these active ingredients may be the key ingredients for GGQLD to exert anti-RVE effects through the above functional pathways. In addition, the serotonin receptor subfamily (HTR1A, HTR2B, and HTR7), the adrenergic receptor subfamily (ADRA2A, ADRA2B, ADRB1, ADRB2, and ADRB3), the cholinergic receptor subfamily (CHRM1, CHRM2, and CHRM3), and the dopamine receptor subfamily (DRD1, DRD2, DRD3, DRD4, and DRD5), respectively, corresponded to 8, 7, 7, and 7 active ingredients. It is speculated that these targets may be the key parts for GGQLD to exert anti-RVE efficacy through the above functional pathways.

### 3.5. GGQLD Inhibited the Release of Calcium Ions in Caco-2 Cells after RV Infection

**Figure 5:**
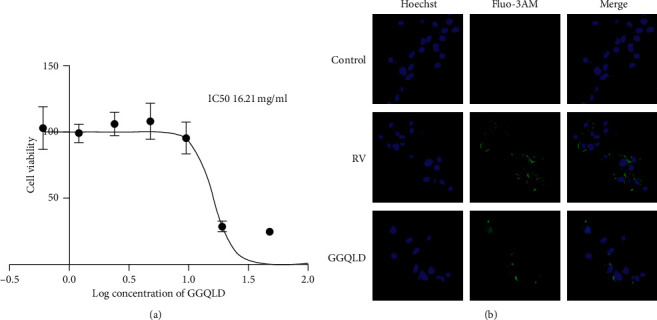
Intracellular calcium ion intensity of Caco-2 cells: (a) the IC50 of GGQLD to Caco-2 cells; (b) the calcium ion intensity of the control group, RV group, and GGQLD group. Blue fluorescence represents the nucleus, and the green fluorescence represents the calcium ions.

After applying 4.8 mg/ml of GGQLD (IC50: 16.21 mg/ml, as shown in Figure 5(a)) to RV infected Caco-2 cells 24 hours, we observe the result with laser confocal microscope. As shown in Figure 5(b), intracellular calcium ion fluorescence intensity in Caco-2 cells infected with the RV was significantly stronger than the control group. However, the GGQLD group could reduce the fluorescence intensity of intracellular calcium ions in RV-infected Caco-2 cells, which showed that GGQLD had access to decrease the release of intracellular calcium ions.

## 4. Discussion

### 4.1. Analysis of Active Ingredients of GGQLD

From the TCMSP database, 130 active ingredients of GGQLD were found to be related to RVE, which mainly included the types of flavonoids, alkaloids, phenyl ester, and fatty acids. The flavonoids have the abilities to destroy the integrity of RV structure and protein synthesis, which has the potentials of anti-RV drug research [18, 19]. An ingredient called licocoumarone, for example, has the ability to suppress viral RNA synthesis in TF-104 cells [20]. From Figure 4, we also found that flavonoids such as formononetin, acacetin, wogonin, and quercetin were distributed in key pathways and target multiple disease genes. In addition, other active ingredients named (R)-canadine, icos-5-enoic-acid, moupinamide, and bis[(2S)-2-ethylhexyl]-benzene-1,2-dicarboxylate also had potential research value. Overall, we could find that Scutellaria baicalensis was the most influential Chinese medicine, containing a variety of key active ingredients.

### 4.2. Closely Related Target Analysis from the PPI Network

The PPI network showed that the deeper potential targets of GGQLD were related to proliferation and transcription. As the histone acetyltransferase, EP300 and KAT2B play a causative role in regulating transcription through internal lysine acetylation of multiple proteins and a rapid and reversible regulatory mechanism [21]. In addition, HSP90AA1, respectively, promotes autophagy and inhibits apoptosis through the PI3K/Akt/mTOR pathway and JNK/P38 pathway [22]. AKT1 (a downstream target of phosphatidylinositol 3-kinase) and CTNNB1 (a central part in Wnt/beta-catenin pathway) both have access to regulate a number of cellular proliferation and differentiation [23, 24]. And with the active autophosphorylation of receptor tyrosine kinase, EGFR could initiate a cascade of downstream signaling pathways involved in regulating cellular differentiation [25]. As for CDK1, it is required for the transition from the G2 phase into mitosis, which affects cell proliferation a lot [26]. In short, these targets with high connectivity are closely related to cell proliferation and differentiation.

### 4.3. The Impact of Hormone Binding

**Figure 6:**
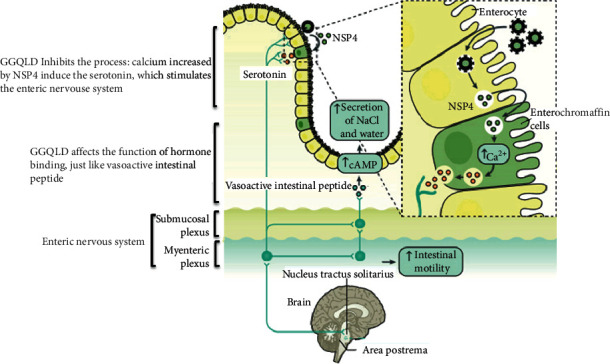
The hypothetical mechanism of GGQLD in treating RVE. First, GGQLD reduced the release of calcium ions from intestinal cells caused by RV, thereby inhibiting the secretion of serotonin. It could impede the impulse of the nervous system of the intestine to reduce the promotion effect of vasoactive intestinal peptide on cAMP, which finally balanced the intestinal water and salt metabolism and eased RVE diarrhea.

Gastrointestinal hormones are the basic regulators of intestinal absorption, metabolism, and homeostasis and play important roles in intestinal fitness [27]. Similar to GGQLD for damp-heat syndrome of TCM, ZHIXIE decoction may improve the efficacy of damp-heat RV infection enteritis by regulating gastrointestinal hormones [28]. In a variety of gastrointestinal hormones, vasoactive intestinal peptide in gastrointestinal hormones is associated with the pathways described below. In general, vasoactive intestinal peptide is synthesized under the activation of serotonin receptors on intrinsic primary afferent nerves that compose the myenteric plexus [29], which can cause diarrhea through up-regulated cAMP [30]. Interestingly, as shown in Figure 6, recent studies have reported that serotonin homeostasis can be destroyed by RV infection [31, 32].

### 4.4. The Analysis of Neuroactive Ligand-Receptor Interaction, Serotonergic Synapse, and Calcium Signaling Pathway

From the perspective of disease, RVE seems to be related to the intestinal nervous system [33]. For example, the enteric neurons have a lot to do with gastrointestinal functions and age, which are also related to RV [34]. And as described in the hormone binding analysis, recent studies have demonstrated that RVE has potential mechanisms that enteric nervous system was affected by serotonin, which was corresponding to the results of neuroactive ligand-receptor interaction and serotonergic synapse obtained by analysis [32]. Furthermore, it also shown that increased intracellular calcium level mediated by NSP4 could induce the serotonin of enterochromaffin cells, which can stimulate the intestinal nervous system and ultimately enhance the irritable bowel movement [31, 35, 36]. Besides, we found that RV infection in infants and young children would disrupt calcium homeostasis to increased calcium ion concentration in intestinal epithelial cells, which was closely related to infection [37]. It was corresponding to the results of calcium ion signaling pathway, which indicated that GGQLD may act on the calcium ion channel to achieve therapeutic effect. In terms of experimental results, this study also preliminarily found that GGQLD could inhibit the condition that RV increased calcium release in Caco-2 cells. In short, we supposed GGQLD mainly achieves antidiarrhea effects on the calcium ion release, serotonin homeostasis, gastrointestinal hormones, and intestinal nerve system (Figure 6).

## 5. Conclusions

GGQLD had 130 active ingredients in the treatment of RVE, such as (R)-canadine, formononetin, and moupinamide. They targeted 366 genes to regulate the secretion of gastrointestinal hormones of intestinal epithelial cells and stabilize the secretion of calcium ions and serotonin, among which serotonin receptors, adrenergic receptors, cholinergic receptors, and dopamine receptors were potential key points. In summary, this study reveals the anti-RVE mechanism of GGQLD through multicomponent-multitarget-multipathway and provided new ideas for comprehensive and in-depth clarification of the mechanism of GGQLD in the treatment of RVE.

## Figures and Tables

**Table 1:** The active ingredients of GGQLD in TCMSP database (their targets intersected with enteritis genes).

Herbs	Compound name	OB	DL	PubChem ID
Pueraria lobate	Formononetin	69.67	0.21	5280378
	3′-Methoxydaidzein	48.57	0.24	5319422
	Daidzein-4,7-diglucoside	47.27	0.67	171292

Scutellaria baicalensis	Acacetin	34.97	0.24	5280442
	Wogonin	30.68	0.23	5281703
	(2R)-7-Hydroxy-5-methoxy-2-phenyl-2,3-dihydrochromen-4-one	55.23	0.2	821279
	Baicalein	33.52	0.21	5281605
	5,8,2′-Trihydroxy-7-methoxyflavone	37.01	0.27	156992
	5,7,3′,6′-Tetrahydroxy-6,8,2′-trimethoxyflavone	33.82	0.45	44258628
	Carthamidin	41.15	0.24	188308
	2,6,2′,4′-Tetrahydroxy-6′-methoxychalcone	69.04	0.22	78385588
	Dihydrobaicalin_qt	40.04	0.21	14135323
	Eriodyctiol	41.35	0.24	373261
	Salvigenin	49.07	0.33	161271
	5,2′,6′-Trihydroxy-7,8-dimethoxyflavone	45.05	0.33	5322059
	5,7,2′,6′-Tetrahydroxyflavone	37.01	0.24	5321865
	Dihydrooroxylin A	38.72	0.23	177032
	Skullcapflavone II	69.51	0.44	124211
	Oroxylin A	41.37	0.23	5320315
	Panicolin	76.26	0.29	5320399
	5,7,4′-Trihydroxy-8-methoxyflavone	36.56	0.27	5322078
	NEOBAICALEIN	104.34	0.44	124211
	2*β*-Phenyl-2,3-dihydro-5,7-dihydroxy-6-methoxy-4H-1-benzopyran-4-one	66.06	0.23	25721350
	*β*-Sitosterol	36.91	0.75	222284
	3-Epi-*β*-sitosterol	36.91	0.75	12303645
	Norwogonin	39.4	0.21	5281674
	5,2′-Dihydroxy-6,7,8-trimethoxyflavone	31.71	0.35	159029
	Ent-Epicatechin	48.96	0.24	182232
	Stigmasterol	43.83	0.76	5280794
	Coptisine	30.67	0.86	72322
	Bis[(2S)-2-ethylhexyl]benzene-1,2-dicarboxylate	43.59	0.35	7057920
	Supraene	33.55	0.42	638072
	Diop	43.59	0.39	33934
	Epiberberine	43.09	0.78	160876
	Moslosooflavone	44.09	0.25	188316
	11,13-Eicosadienoic acid, methyl ester	39.28	0.23	5365674
	5,7,4′-Trihydroxy-6-methoxyflavanone	36.63	0.27	5322074
	5,7,4′-Trihydroxy-8-methoxyflavanone	74.24	0.26	42608119
	Rivularin	37.94	0.37	13889022

Coptis chinensis	Berberine	36.86	0.78	2353
	Obacunone	43.29	0.77	119041
	Berberrubine	35.74	0.73	72703
	(R)-Canadine	55.37	0.77	443422
	Berlambine	36.68	0.82	11066
	AC1O7GAS	104.95	0.78	6604198
	Magnograndiolide	63.71	0.19	5319198
	Palmidin A	35.36	0.65	5320384
	Palmatine	64.6	0.65	19009
	Quercetin	46.43	0.28	5280343
	Worenine	45.83	0.87	20055073
	Moupinamide	86.71	0.26	5280537
Glycyrrhiza uralensis	Inermine	75.18	0.54	91510
	DFV	32.76	0.18	114829
	Mairin	55.38	0.78	64971
	Glycyrol	90.78	0.67	5320083
	Jaranol	50.83	0.29	5318869
	Medicarpin	49.22	0.34	336327
	Isorhamnetin	49.6	0.31	5281654
	3-Epi-*β*-sitosterol	36.91	0.75	12303645
	Lupiwighteone	51.64	0.37	5317480
	7-Methoxy-2-methyl isoflavone	42.56	0.2	911486
	Formononetin	69.67	0.21	5280378
	Calycosin	47.75	0.24	5280448
	Kaempferol	41.88	0.24	5280863
	Naringenin	59.29	0.21	932
	Shinflavanone	31.79	0.72	197678
	Glyasperin B	65.22	0.44	480784
	Glyasperin F	75.84	0.54	392442
	Glyasperin C	45.56	0.4	480859
	Isotrifoliol	31.94	0.42	5318679
	(E)-1-(2,4-Dihydroxyphenyl)-3-(2,2-dimethylchromen-6-yl)prop-2-en-1-one	39.62	0.35	10881804
	Kanzonol W	50.48	0.52	15380912
	(2S)-6-(2,4-Dihydroxyphenyl)-2-(2-hydroxypropan-2-yl)-4-methoxy-2,3-dihydrofuro[3,2-g]chromen-7-one	60.25	0.63	637112
	Semilicoisoflavone B	48.78	0.55	5481948
	Glepidotin A	44.72	0.35	5281619
	Glepidotin B	64.46	0.34	442411
	Phaseolinisoflavan	32.01	0.45	4484952
	Glypallichalcone	61.6	0.19	5317768
	8-(6-Hydroxy-2-benzofuranyl)-2,2-dimethyl-5-chromenol	58.44	0.38	10542808
	Licochalcone B	76.76	0.19	5318999
	Licochalcone G	49.25	0.32	49856081
	Licoarylcoumarin	59.62	0.43	10090416
	Licoricone	63.58	0.47	5319013
	Gancaonin A	51.08	0.4	5317478
	Gancaonin B	48.79	0.45	5317479
	Licorice glycoside E	32.89	0.27	42607811
	Gancaonin L	66.37	0.41	14604077
	Gancaonin M	30.49	0.41	14604078
	Gancaonin O	44.15	0.41	14604081
	Glycyrin	52.61	0.47	480787
	Licocoumarone	33.21	0.36	503731
	Licoisoflavone	41.61	0.42	5281789
	Licoisoflavone B	38.93	0.55	5481234
	Licoisoflavanone	52.47	0.54	392443
	Shinpterocarpin	80.3	0.73	10336244
	(E)-3-[3,4-Dihydroxy-5-(3-methylbut-2-enyl)phenyl]-1-(2,4-dihydroxyphenyl)prop-2-en-1-one	46.27	0.31	11267805
	Liquiritin	65.69	0.74	503737
	Licopyranocoumarin	80.36	0.65	122851
	3,22-Dihydroxy-11-oxo-delta(12)-oleanene-27-alpha-methoxycarbonyl-29-oic acid	34.32	0.55	195396
	Glyzaglabrin	61.07	0.35	5317777
	Glabridin	53.25	0.47	124052
	Glabranin	52.9	0.31	124049
	Glabrene	46.27	0.44	480774
	Glabrone	52.51	0.5	5317652
	Hedysarimcoumestan B	48.14	0.43	11558452
	3,5-Dihydroxy-13,14-dimethoxy-8,17-dioxatetracyclo[8.7.0.0(2),.0(1) (1),(1)]hepta-deca-1(10),2,4,6,11,13,15-hepta-en-9-one	62.9	0.53	1160239
	Eurycarpin A	43.28	0.37	5317300
	Glycyroside	37.25	0.79	44257223
	(-)-Medicocarpin	40.99	0.95	23724664
	Sigmoidin-B	34.88	0.41	73205
	(2R)-7-Hydroxy-2-(4-hydroxyphenyl)-2,3-dihydrochromen-4-one	71.12	0.18	928837
	Isobavachin	36.57	0.32	193679
	Isoglycyrol	44.7	0.84	124050
	Isolicoflavonol	45.17	0.42	5318585
	Isoformononetin	38.37	0.21	3764
	1-Methoxyphaseollidin	69.98	0.64	480873
	Quercetin der	46.45	0.33	52761906
	3′-Hydroxy-4′-O-methylglabridin	43.71	0.57	15228662
	Licochalcone A	40.79	0.29	5318998
	3′-Methoxyglabridin	46.16	0.57	15228663
	4′-Methoxyglabridin	36.21	0.52	9927807
	Icos-5-enoic acid	30.7	0.2	3349565
	Kanzonol F	32.47	0.89	101666840
	6-Prenylated eriodictyol	39.22	0.41	13845972
	7,2′,4′-Trihydroxy-5-methoxy-3-arylcoumarin	83.71	0.27	25015742
	7-Acetoxy-2-methylisoflavone	38.92	0.26	268208
	(2S)-3′,4′,5,7-Tetrahydroxy-8-prenylflavanone	53.79	0.4	13845973
	Gadelaidic acid	30.7	0.2	5460988
	Vestitol	74.66	0.21	92503
	Gancaonin G	60.44	0.39	480780
	Gancaonin H	50.1	0.78	5481949
	Licoagrocarpin	58.81	0.58	15840593
	Glyasperin M	72.67	0.59	101664572
	Glycyrrhiza flavonol A	41.28	0.6	5317765
	Licoagroisoflavone	57.28	0.49	636883
	18-*α*-Hydroxyglycyrrhetic acid	41.16	0.71	101280181
	Odoratin	49.95	0.3	13965473
	Phaseol	78.77	0.58	44257530
	Xambioona	54.85	0.87	14769500
	Dehydroglyasperin c	53.82	0.37	480775
	Quercetin	46.43	0.28	5280343

**Table 2:** Names and PubChem IDs of GGQLD active ingredients (number of intersecting targets >5).

Herbs	Compound name	PubChem ID	OB	DL
Pueraria lobate	3′-Methoxydaidzein	5319422	48.57	0.24
	Daidzein-4,7-diglucoside	171292	47.27	0.67
	Acacetin	5280442	34.97	0.24

Scutellaria baicalensis	Wogonin	5281703	30.68	0.23
	(2R)-7-Hydroxy-5-methoxy-2-phenyl-2,3-dihydrochromen-4-one	821279	55.23	0.2
	Baicalein	5281605	33.52	0.21
	5,8,2′-Trihydroxy-7-methoxyflavone	156992	37.01	0.27
	2,6,2′,4′-Tetrahydroxy-6′-methoxychalcone	78385588	69.04	0.22
	*β*-Sitosterol	222284	36.91	0.75
	Bis[(2S)-2-ethylhexyl]benzene-1,2-dicarboxylate	7057920	43.59	0.35
	Supraene	638072	33.55	0.42
	Diop	33934	43.59	0.39
	11,13-Eicosadienoic acid methyl ester	5365674	39.28	0.23
	(R)-Canadine	443422	55.37	0.77

Coptis chinensis	Quercetin	5280343	46.43	0.28
	Moupinamide	5280537	86.71	0.26
	Mairin	911486	55.38	0.78
	Formononetin	5280378	69.67	0.21

Glycyrrhiza uralensis	Glypallichalcone	5317768	61.6	0.19
	Icos-5-enoic acid	3349565	30.7	0.2
	7-Acetoxy-2-methylisoflavone	268208	38.92	0.26
	Gadelaidic acid	5460988	30.7	0.2

## Data Availability

The data used to support the findings of this study are available from the corresponding author upon request.
